# Influence of Label Design and Country of Origin Information in Wines on Consumers’ Visual, Sensory, and Emotional Responses

**DOI:** 10.3390/s22062158

**Published:** 2022-03-10

**Authors:** Chang Liu, Chetan Sharma, Qiqi Xu, Claudia Gonzalez Viejo, Sigfredo Fuentes, Damir D. Torrico

**Affiliations:** 1 Centre of Excellence—Food for Future Consumers, Department of Wine, Food and Molecular Biosciences, Faculty of Agriculture and Life Sciences, Lincoln University, Lincoln 7647, New Zealand; yvonne.liu@lincolnuni.ac.nz (C.L.); chetan.sharma@lincoln.ac.nz (C.S.); qiqi.xu@lincolnuni.ac.nz (Q.X.); 2 Digital Agriculture Food and Wine Group, School of Agriculture and Food, Faculty of Veterinary and Agricultural Sciences, University of Melbourne, Parkville, VIC 3010, Australia; cgonzalez2@unimelb.edu.au (C.G.V.); sigfredo.fuentes@unimelb.edu.au (S.F.)

**Keywords:** provenance, logo, wine labels, eye-tracking, consumers’ acceptability

## Abstract

This study aimed to evaluate the influence of origin information on Pinot Noir wine labels using eye-tracking and its associations with purchase intent, and hedonic and subconscious emotional responses. Two studies were carried out on untrained university staff and students aged 20–60 years old. Study 1 was conducted to assess consumers’ (*n* = 55; 55% males, and 45% females) self-reported and subconscious responses towards four design labels (with and without New Zealand origin name/script or origin logo) using eye-tracking and video analysis to evaluate emotions of participants. In study 2, participants (*n* = 72, 56% males, and 44% females) blind-tasted the same wine sample from different labels while recording their self-reported responses. In study 1, no significant differences were found in fixations between origin name/script and origin logo. However, participants paid more attention to the image and the brand name on the wine labels. In study 2, no significant effects on emotional responses were found with or without the origin name/script or logo. Nonetheless, a multiple factor analysis showed either negative or no associations between the baseline (wine with no label) and the samples showing the different labels, even though the taste of the wine samples was the same, which confirmed an influence of the label on the wine appreciation. Among results from studies 1 and 2, origin information affected the purchase intent and hedonic responses marginally. These findings can be used to design wine labels for e-commerce.

## 1. Introduction

The country-of-origin (COO) is a century-old trade strategy wherein merchants of distant lands started exchanging goods with ecological diversity [1]. However, it became a trend only in recent times under the manifestations of the so-called post-consumerism, which occurred in the aftermath of the commodification of food production systems and consumption. Nowadays, COO is being pushed, in an environmental fact form [2], by both governments [3,4] and businesses for various reasons. COO can be used not only as a factor that affects the rating or acceptability of consumers but also as an indication of product quality, perceived risk, and the likelihood of purchase [5]. Research shows that origin information affects the purchase decision [4], depending on the stereotypes formed concerning the country-of-origin [6,7]. Consequently, a positive image of the country can highlight the relationship between the product and its origin.

Wine, currently at the height of attention, identification [8,9], and imagination [10], has been historically associated with a certain place or terroir, such as Burgundy and Bordeaux. Raised on the concept of ‘taste of place’, wine is extensively sold and bought based on its place-based identity. Previously, wineries, from both the “Old” and “New” worlds, frequently employed COO as an influencing factor to snatch as much share of the market as possible [5]. This is why wine industries around the world have put a lot of effort into highlighting the country-of-origin information on wine labels over the last few decades.

Consumers are now becoming more attracted to extrinsic cues from premium products. For instance, price and wine region were the most important extrinsic factors for consumers when evaluating Australian wines [11]. However, the mere labeling of COO does not guarantee success in the market because top-down and bottom-up mechanisms may influence gaze behavior, attention, and decision-making. Top-down factors are related to previous concepts or ideas about the product that consumers have, and bottom-up factors are characteristics of the stimuli that affect consumer behaviors [12]. The provisioning of day-to-day foods and beverages involves conscious and unconscious visual inspections of labels for the brand, color, character, nutrition, or other features, and most of the time, this gazing behavior involves both top-down and bottom-up processes during the decision-making [13]. Increasingly, research efforts were recorded recently where eye-tracking was used, for instance, to assess nutrition label usage [14], wine labels design [15] and purchase intention [16], emotions [17], restaurant menus [18], and other related uses [19]. Other examples of the use of eye-tracking include the modification of labels to improve the consumer understanding of the information that is presented. Color-coded labels (traffic light systems showing the importance of nutrients using colors) were studied using eye-tracking [20].

Hence, this present study was conducted to assess the effect of “New Zealand placed wines” on consumers’ purchasing decisions and sensory perceptions using novel eye-tracking and traditional sensory techniques from the COO notion. This experiment aimed to understand the effects of COO on emotions, sensory perceptions, overall liking, and purchase decisions. To achieve this, gaze behavior was assessed using an eye-tracking device; furthermore, these eye-tracking results were compared with the sensory responses from wine tasting.

## 2. Materials and Methods

### 2.1. Participants

**Figure 1:**
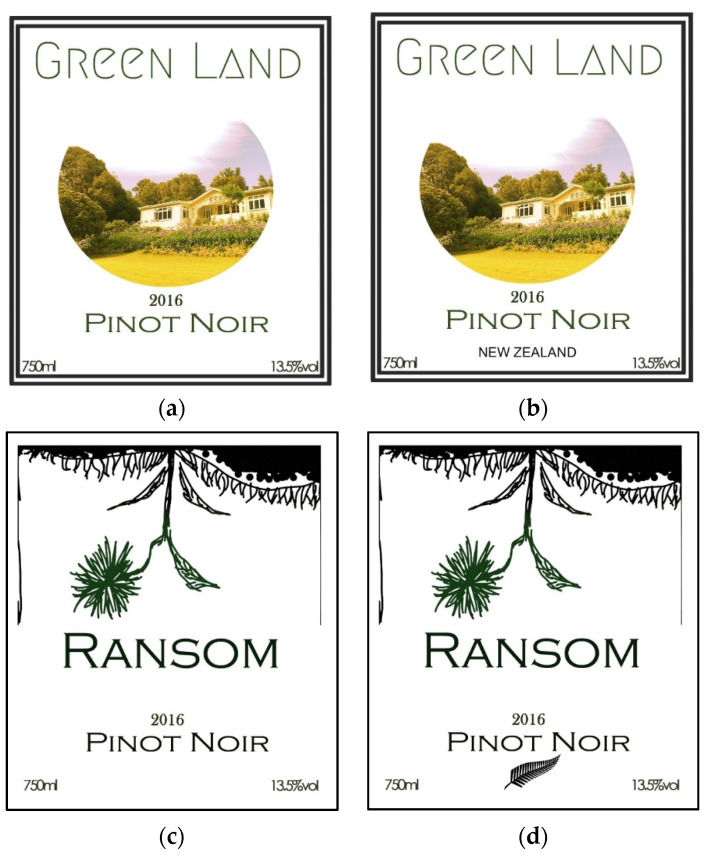
Wine label types with and without provenance emblem (New Zealand and 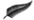) used for this study. Four labels were tested: (**a**,**b**) had the same design, but one included a piece of COO information in the textual form “New Zealand” (**b**), whereas the other did not (**a**). Similarly, (**c**,**d**) had a matching design (different from (**a**,**b**)), but one included COO information in the form of a symbol, i.e., fern—a well-known logo in New Zealand (**d**), whereas the other did not (**c**).

This research was approved by the Human Ethics Committee (HEC—2019-68) of Lincoln University, NZ, and participants were asked to provide their signed consent. The study was conducted in two parts. Study 1 involved evaluating the labels using eye-tracking techniques, while study 2 involved an affective-level investigation using traditional questionnaire settings. Both studies were conducted in sensory booths equipped with standard fluorescent lights at room temperature (25 °C). For both studies, all participants were aged 20–60 years old and recruited via email from Lincoln University, New Zealand. Participants were pre-screened to filter those that were at least occasional wine consumers. Study 1 had *n* = 55 participants (71% Asians, 22% Oceanians; 55% males, 45% females) and were randomly separated into two groups, namely group A (*n* = 27) and B (*n* = 28). This was carried out to avoid carryover effects by presenting similar pairs of labels to each participant. Group A was shown labels a and d, while group B was shown labels b and c (Figure 1) in a random order. In study 2 (tasting), *n* = 72 consumers (63% Asians, 21% Oceanians; 56% males and 44% females) participated.

### 2.2. Task Design and Stimuli

Among the four label stimuli presented in this study, Figure 1a,b had the same design, but one included a piece of COO information in the textual form “New Zealand” (Figure 1b), while the other did not (Figure 1a). Similarly, (Figure 1c,d) had a matching design (different from (Figure 1a,b)), but one included COO information in the form of a symbol, e.g., fern—a well-known logo in New Zealand [21] (Figure 1d), while the other did not (Figure 1c).

GP3 eye tracker (Model—GP3-19034467, Gazepoint, Vancouver, BC, Canada) and iMotions software (iMotions, Denmark), were used together to digitally obtain and process eye fixation measures and facial expressions of participants. The GP3, a portable (1.4 kg) non-intrusive eye tracker (0.5–1.0 degrees of visual angle accuracy), was coupled to a 19” monitor (DELL P1913 Monitor with LED, DELL, Round Rock, TX, USA) with a maximum resolution of pixels (1366 × 768) and a refresh rate of 60 Hz. Seven areas of interest (AOI) were tested in each label including, (i) alcohol (%) information, (ii) country of origin (COO) information, (iii) brand name, (iv) photo (central figure), (v) type of wine information, (vi) volume, and (vii) year (vintage). For each label, the parameters obtained from eye-tracking were (i) time to first fixation (TFF)—the time from starting the test until the start of the first fixation in an AOI (ms), (ii) first fixation duration—duration of the first individual fixation within an AOI (ms), (iii) average fixation duration—an average of the fixation duration for each AOI (ms), and (iv) average fixation count—number of fixations or number of times the participants viewed a certain position within an AOI during the session. The calculation of the average fixation count involves the summation of all fixation counts (for all participants) divided by the number of participants in a particular group. For instance, if the average fixation value is 0.5, and the number of participants for that group is 28, the total fixation count would be 0.5 × 28 = 14. In line with the standard methods of visualizing eye-tracking data, heat-map graphics were obtained, in which the accumulated fixations of participants on each area of the stimulus were plotted. Important tools for analyzing eye-movement data are the areas of interest (AOIs), which define important regions of the label.

To analyze emotions using biometrics, the iMotions facial expression analysis module was used; this software uses the algorithms from Affectiva (Affectiva, Boston, MA, USA) [22] and Realeyes (Realeyes, London, UK) [23], which work using computer vision for face detection, recognition and tracking, and machine learning algorithms to associate facial expressions with specific emotions. iMotion detects and extracts seven core emotions (joy, anger, fear, disgust, contempt, sadness, and surprise) and 20 facial expression measurements [24]. Participants were asked to sit comfortably in front of the monitor and perform the calibration task (following 9 points on the screen with their sight) using iMotions. They were then instructed to look at the first tested label (out of two possible labels) shown on the screen for 10 s and asked to evaluate the label by answering questions related to its acceptability. The overall liking was assessed using a 9-point hedonic scale (1 = Dislike extremely, 5 = Neither like nor dislike, 9 = Like extremely). The purchase intention was included as a binary “yes/no” question. This was repeated for a second sample. Once both labels were evaluated, the importance of label elements (logo, brand name, origin, type of wine, and year) in the purchase decision was determined on a 9-point scale (1 = Not at all, 5 = Neutral, 9 = Extremely important). Demographic questions regarding wine-drinking frequency (1 = Every day, 2 = Every week, 3 = Once every two weeks, 4 = Once a month, 5 = Occasionally), gender, age, and nationality were also included.

**Figure 2:**
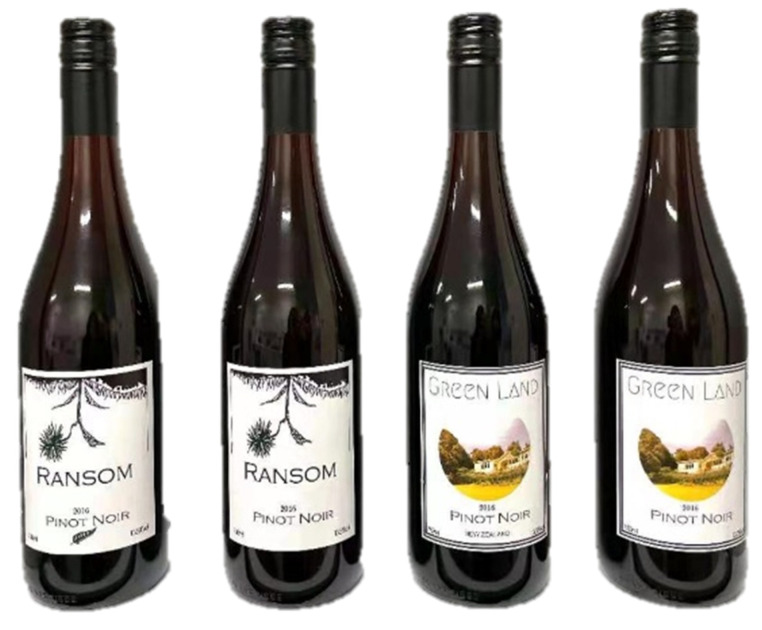
Wine bottles with manipulated labels showed in study 2.

For study 2, the aforementioned labels were used to replicate an actual condition type. The labels were placed on wine bottles (Figure 2) to mimic the actual wine condition sold in the market. A wine of Akarua Winery Ltd.,—Rua Pinot Noir (Akarua Ltd., Cairnmuir Road, Bannockburn, Central Otago, New Zealand) was used. All participants were served one tray with three glasses simultaneously. Each glass (Spiegelau # 440 01 31) contained 35 mL of wine. The wine was poured 30 min before tasting at room temperature (21 °C). Each tray contained a glass of wine and a labelled bottle to make an informed evaluation. A randomized order was used to minimize the order bias. The participants were asked to taste and evaluate the wines with bottles (with different labels) and the control wine (no bottle was provided) without being aware that all samples had the same wine (Rua Pinot Noir). Similar to study 1, in study 2, participants were randomly separated into two groups: group A (*n* = 36) and B (*n* = 36). Group A was shown wines with labels a and d (plus a blind control sample without showing any bottle), while group B was shown labels b and c (plus a blind control sample without showing any bottle). Study 2 was also divided into two groups since the label pairs were too similar to be tested on participants in one setting. Having similar labels could produce carryover effects, which might hinder some of the responses. A cross-over design with a random assignation of participants to each session group was implemented in this study.

Participants were asked to taste the wines and answer the questions of acceptability, perception and emotions using the RedJade^®^ Sensory software (Redjade^®^, Martinez, CA, USA) on the tablet. A total of 16 emotions, including happy, neutral, sad, curious, disgusted, surprised, excited, pleased, calmed, apprehensive, comforted, satisfied, bored, guilty, healthy, and unhealthy, were provided to participants, who were asked to check-all-that-apply (CATA) for each the sample. Terms were selected based on a previous study in emotional responses towards wine products [25]. Likewise, participants were asked to provide information on the sensory profile of wines using CATA, which had options of processed fruit aroma, sweet aroma, green aroma, spicy aroma, earthy aroma, chemical aroma, heat feeling, sweet taste, bitter taste, and astringent. The overall liking of the wines was also included using a 9-point hedonic scale (1 = Dislike extremely, 5 = Neither like nor dislike, 9 = Like extremely). Similar to study 1, purchase intent was added as a binary scale (yes/no).

The participants were instructed to have a 30 s break between samples and cleanse their palate thoroughly using plain water and water crackers.

### 2.3. Statistical Analysis

Data from overall liking, the importance of label elements on purchasing decision analysis, eye fixation, and emotions were analyzed by analysis of Variance (ANOVA) and Tukey’s test (α = 0.05) using Minitab 18.1 (Minitab, Inc., 1829 Pine Hall Rd, State College, PA, USA). In study 2, Cochran’s Q test was used to analyze the result of sensory CATA and emotion CATA by using XLSTAT 2019.4.2 (Addinsoft, New York, NY, USA). Multivariate data analysis was conducted for both studies. In study 1, a principal components analysis (PCA) was developed using the self-reported and subconscious emotional responses using a code written in Matlab^®^ R2021a (Mathworks, Inc., Natick, MA, USA) to assess relationships and associations between variables and samples. On the other hand, for study 2, a multiple factor analysis (MFA) was conducted using quantitative (self-reported liking) and frequency data (CATA emotions and sensory descriptors) using XLSTAT.

## 3. Results and Discussion

### 3.1. Study 1: Wine Labels

#### 3.1.1. Eye-Tracking on Areas of Interest (AOI)

The COO information on the label with a script produced a significant (*p* < 0.05) lower time of first fixation (TFF, in ms) compared to that of the label with no script (Table 1). On the other hand, the TFF of the COO information was similar for labels with and without logos. The TFF, taken by logo over script type, was lower but not significant (*p* ≥ 0.05). Comparing the logo or script with a blank space measured the signal of that particular stimulus against the noise of not having an element in the label. The results can be interpreted in such a way that, if significant differences are found between the signal and noise, the presence of that particular element triggers a higher response on the eye-tracking measurements. This information may have important practical implications for wineries and other industries, as companies consciously design labels to decrease the time it takes participants to first view the area of interest. The lowest TFF was reported for the photo section of the label, irrespective of the label type, while the highest TFF was reported for the volume information in all label types except for the no script label. A hierarchy of gaze behaviors can be extracted from Table 1, where the photo (central figure in the label) grabs the attention first, followed by the brand name and type of wine. Pictures were previously reported to cause quicker gazing reactions than words. This effect is known as the picture superiority effect [26]. Moreover, the position bias (center) of the photo, especially in labels a (no script) and b (script), could be another reason for explaining the lowest time that observers took to make the first fixation; it is reported elsewhere that observers show a marked tendency to fixate at the center of the screen, independent of image features [27]. Underpinning this, Gofman et al. [28] reported that the fixation path typically starts in the middle of the image on the wine box. Nevertheless, COO information is not an eye-catching element at first glance, but this has certainly an afterthought influence compared to no COO information when evaluating the label as a whole.

**Figure 3:**
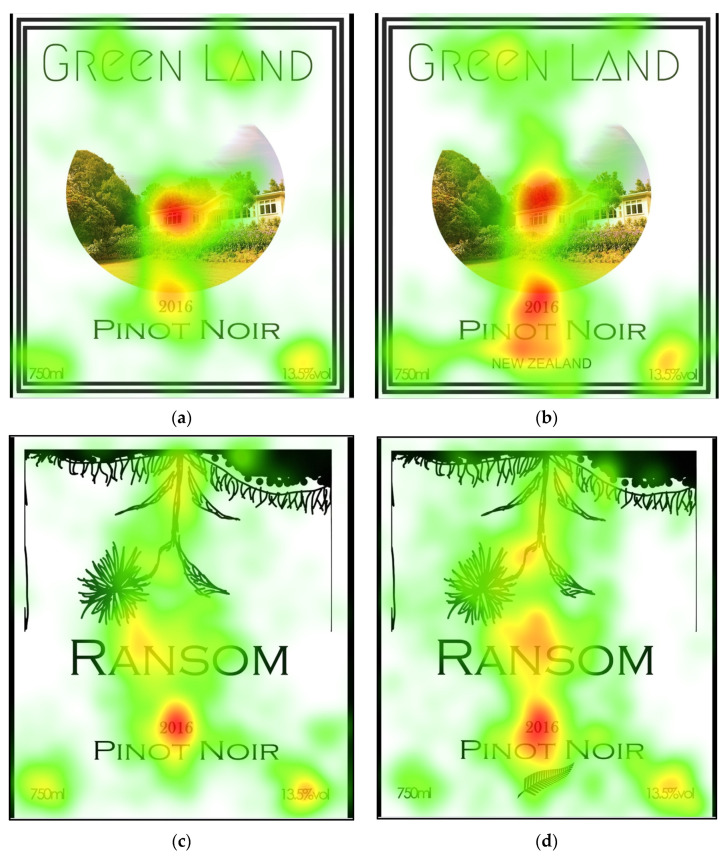
A heat map representation of the labels used for Study 1. Four labels were tested in this study: (**a**,**b**) had the same design, but one included a piece of COO information, in the textual form, “New Zealand” (**b**), whereas the other did not (**a**). Similarly, (**c**,**d**) had a matching design (different from (**a**,**b**)), but one included COO information in the form of a symbol, i.e., fern—a well-known logo in New Zealand (**d**), whereas the other did not (**c**).

Concerning relative engagement with the COO information, irrespective of the script or logo form, no significant (*p* > 0.05) differences were observed (Table 2). Similar to the TFF trend, participants’ gaze was relatively still for a longer time on the photo, followed by the brand and type of wine information (Figure 3). Moreover, a longer engagement was seen with logotype over the script regarding the first fixation duration. Ultimately, longer engagements affect persuasion and lead to more favorable brand attitudes. Logos were previously noted to capture and transfer attention to other elements of the label [29].

For the average fixation duration (ms) of the labels (Table 3), the volume AOI for the logo label had a significantly lower time than the other labels. This might be partially explained because the logo (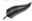) and year information captured most of the fixation time in that region (lower part) of the label. On other hand, the COO in the no script label had the lowest average fixation duration time compared to those of the other labels. In terms of the different AOI, the photo had the highest average fixation duration compared to the other elements. Since the photo had the highest area in the label and was located in the center, participants spent more time looking at this element compared to the other AOIs in the labels.

On average, the photo was more engaging than any other AOIs (Table 3). Simultaneously, COO information as a script was more engaging than no COO information. A similar trend was observed with the logo and no logo labels, but this difference was non-significant (*p* ≥ 0.05). This could be attributed to the attention transferring feature of the logos [29]. A supporting visual of this phenomenon can be observed in the heat map (Figure 3). Logos were previously reported to transfer attention to the other elements of labels [29].

A similar trend was reported for fixation counts (Table 4), which measures the number of fixations within each AOI. No significant differences (*p* ≥ 0.05) were found among the treatment labels in this parameter for all AOIs. Moreover, the photo AOI had the highest fixation counts compared to the other elements. These results concur with the findings observed in the first fixation duration (Table 2) and average fixation duration (Table 3), highlighting the importance of the photo as the central element of the label.

#### 3.1.2. Multivariate Data Analysis

**Figure 4:**
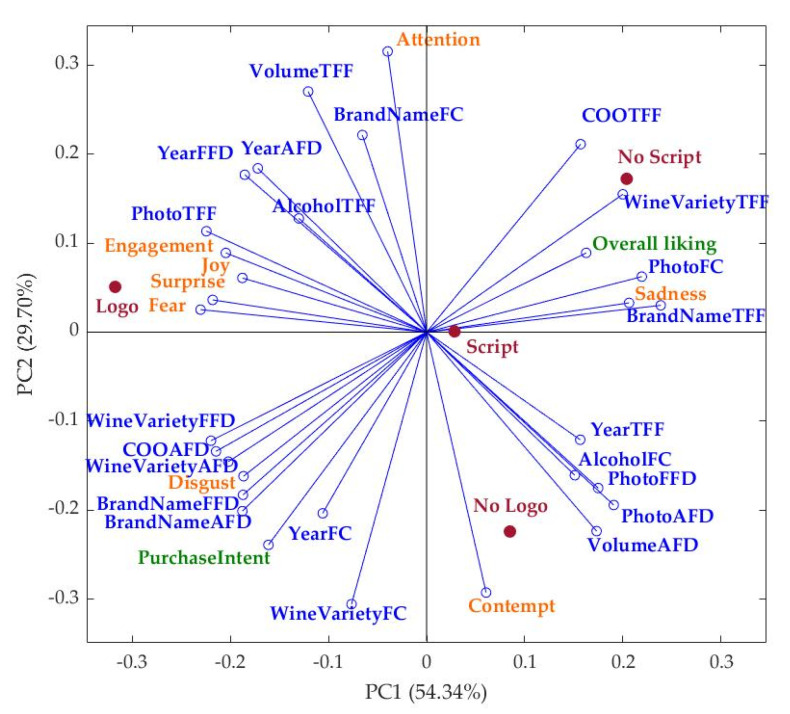
Principal component analysis (PCA) of the biometrics and self-reported responses obtained from study 1 for wine labels. Variables in blue belong to the eye-tracking measurements, orange represents the emotional responses and green represents self-reported responses. Abbreviations: PC: principal component; COO: country of origin; FC: fixation count; TFF: time to the first fixation; FFD: first fixation duration; AFD: average fixation duration.

Figure 4 shows that principal component one (PC1) represented 54.34% of data variability, while PC2 accounted for 29.70% (total PC: 84.04%). According to the factor loadings (FL), PC1 was mainly represented on the positive side of the axis by BrandNameTFF (FL = 0.24), PhotoFC (FL = 0.22), and Sadness (FL = 0.21), and on the negative side by Fear (FL = −0.23), Surprise (FL = −0.22), PhotoTFF (FL = −0.22), and WineVarietyFFD. On the other hand, PC2 was mainly represented by Attention (FL = 0.32) and VolumeTFF (FL = 0.27) on the positive side of the axis and by WineVarietyFC (FL = −0.31) and Contempt (FL = −0.29) on the negative side. It can be observed that the overall liking of the label was positively related to WineVarietyTFF and PhotoFC and negatively related to Disgust and the average fixation duration of COO, WineVariety, and BrandName. Furthermore, attention had a positive relationship with BrandNameFC and Volume TFF and a negative relationship with Contempt. On the other hand, the label with the logo was mainly associated with emotions such as engagement, joy, surprise and fear, while the label with no logo was associated with Contempt and VolumeAFD. The script label was located in the center of the PCA with a slightly higher association with Sadness and BrandNameTFF. Moreover, the no script label was related to COOTFF, WineVarietyTFF and overall liking of the label, with a negative association with purchase intention.

Hessels et al. [30] indicated that the shape, size, and location of AOI elements caused significant variations in eye-tracking responses. In the present study, the higher attention on the photo AOI can be partially explained due to its relative size compared to other AOIs [31]. The year was another interesting AOI in the labels (Figure 3), which showed the vintage of wines. Year AOI had a higher number of fixation counts compared to COO; the possible reason for this could be the labels’ designs [32]. The labels’ viewing patterns usually start from the top to the bottom of the whole area [33]. The year AOI was located in the middle of all labels and close to the photo and brand name AOIs. However, the COO (script or logo) was located on the bottom part of the label. The top-to-bottom viewing pattern could be the reason why more participants gazed at the year AOI compared to that of COO, disregarding the overall theme of the labels.

### 3.2. Study 2: Wine Tasting

From the emotions CATA results (Supplementary Material: Table S1), except for neutral, no other self-reported emotion after tasting the wine sample was found to be statistically significant at α = 0.05. A similar observation was reported in a published study [28], where neutral and calm were the most selected self-reported emotions when consumers looked at wine labels.

Twelve different sensory attributes relevant to wines were provided to participants in the form of CATA (Table S2). Only a few attributes, such as chemical flavor, heat, and sweet taste, were affected by the COO information. The highest perceptual difference concerning chemical flavor and sweet taste was reported between the logo (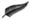) and no logo forms of COO information. The baseline with no label had a significantly higher heat mouthfeel compared to the other four groups with the labels. The heat produced by alcohol is mediated by the pain senses [34], and alcohol percentage could be responsible for this description. Furthermore, no label condition contributed astringency while withholding sweetness (Table S2). No significant differences were found in the other sensory attributes of the wines with different labels. The main purpose of study 2 was to link the consumers’ tasting responses to the emotions and label elements. In doing this, the same wine was used for all the samples that were tasted in the experiment. It was expected to have marginal differences in the taste since the participants tasted the same wine with different labels. Therefore, the analysis of the results does not follow a traditional univariate form when looking at the differences. Further tests must be conducted to understand the connections between taste perception, emotions, label elements and eye-tracking.

**Figure 5:**
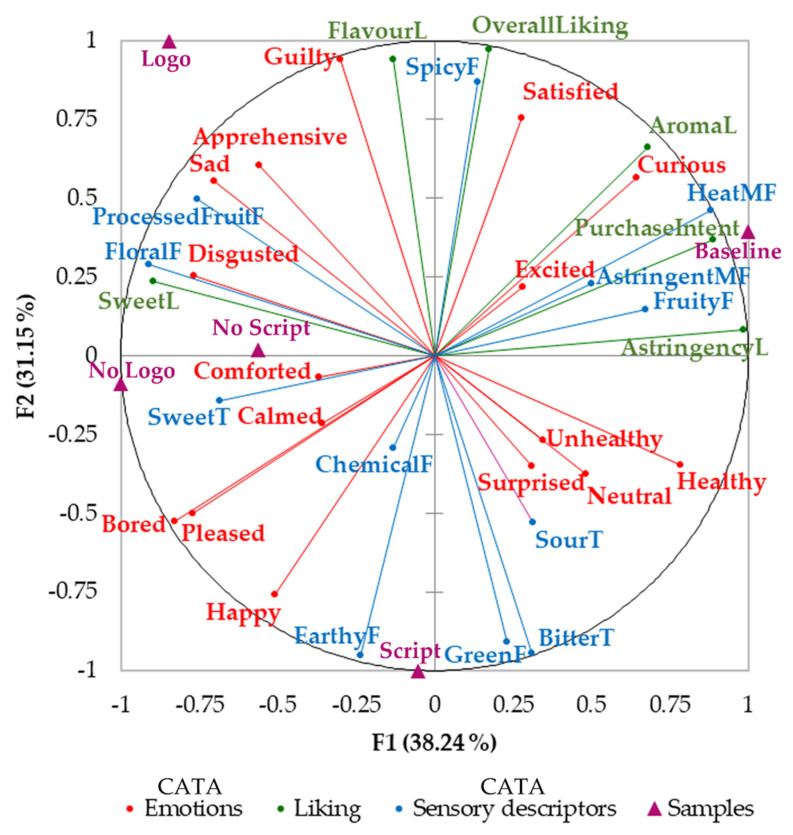
Multiple factor analysis of the check all that apply (CATA) tests for emotions and sensory descriptors and self-reported liking responses. Abbreviations: F: flavor, MF: mouthfeel; L: liking; T: taste.

Figure 5 shows that the factors one and two (F1; F2) of the MFA represented a total of 69.39% of data variability. According to correlation coefficients (r) between the variables and factors, F1 was mainly represented by astringent liking (r = 0.99), and purchase intention (r = 0.89) on the positive side of the axis and floral flavor (r = −0.91) and sweet liking (r = −0.90) on the negative side. On the other hand, F2 was represented on the positive side of the axis by overall liking (r = 0.97), flavor liking (r = 0.94) and guilty (r = 0.94), and on the negative side by an earthy flavor (r = −0.95) and bitter taste (r = −0.95). It can be observed that the baseline sample elicited different emotions, sensory descriptors and purchase intention compared to those when participants saw the label. Baseline was positively associated with purchase intention, heat mouthfeel, excited, astringent mouthfeel, fruity flavor, and astringent liking. Samples with no logo and no script were visually grouped and associated with comforted, sweet taste, floral flavor and disgusted, but negatively associated with attributes from the baseline sample. Moreover, the sample showing the label with script was related to an earthy flavor, green flavor and bitter taste and negatively associated with overall liking. On the other hand, the sample with a logo was associated with guilty, apprehensive, sad, and processed fruit flavor.

When purchasing wine, country-of-origin information and brand familiarity are important factors for consumers [35]. The information labels convey to consumers can affect their appreciation and assessment of wine products. Moreover, the design of the label (including the country of origin information) might also affect the taste perception of consumers [32]. For instance, Veale and Quester [36] used three different wine origin locations (USA, France, and Chile) and found significant differences in taste expectations and quality perception of these products. It is widely recognized that visual attention towards label information is highly related to product choice behaviors [37]. The influence of a product’s origin on consumers’ perceptions was found in previous research [38,39,40]. However, most of these studies centered on self-reported importance based on memory, which can be a poor indicator of consumers’ choices [41]. In addition, the element that consumers pay more attention to does not automatically transform into a higher perception of value and interest in wine products [15]. Thus, cognitive bias could lead to a difference between the data obtained from self-reported behaviors and the actual behaviors of consumers. The difference between unconscious and conscious perceptions is in line with the results of Merdian, Piroth, Rueger-Muck and Raab [15], where significant differences between the unconscious perceptions of wine bottles and conscious purchase decisions were found.

A different study suggested that minimal differences between facial expressions and self-reported emotions existed during the tasting of beverages [42]. On the other hand, Orth et al. [43] reveal that the emotional profile is a significant predictor of wine origin preferences. The physiological signals could be used to monitor the emotional changes in foods and/or beverages on further studies related to the country of origin information [44]. Different information elements on labels have significant influences on emotions [45,46]. The present study showed that visual attention based on gaze fixations was related to the emotions and hedonic responses of consumers towards wine labels.

## 4. Conclusions

Irrespective of the label type, photo and brand name reported a lower incidence of attracting the gaze of participants. Moreover, the photo and brand name also had a longer gaze time than other elements. Country-of-origin (COO) information, in the form of logo, fetch more attention over the script or textual form. Logo helped transfer attention to other label elements and may help fluster gaze. The black fern logo was correlated with surprise, fear, joy, and attention expressions. No effect of COO information was found on the self-reported emotions selection. Both top-down and bottom-up mechanisms seem to play a significant role in consumers’ purchase decisions. The findings of this research could be applicable to wine industries in the designing of wine labels and for e-commerce. Moreover, this research proposes an innovative methodology in the sensory evaluation of labels in general.

## Figures and Tables

**Table 1:** Means and standard deviations of the time to first fixation (ms) on each AOI for four labels *.

Area of Interest	COO Information			
	Script	No Script	Logo 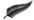	No Logo
Alcohol % ^(NS)^	8873 ^ab^ ± 2198	8557 ^ab^ ± 2520	8785 ^a^ ± 2460	8433 ^a^ ± 2569
COO	8508 ^B,ab^ ± 3309	9961.9 ^A,ab^ ± 833	8440 ^B,a^ ± 3163	8554 ^B,a^ ± 3422
Brand name ^(NS)^	6443 ^bc^ ± 4134	6829 ^b^ ± 3945	5714 ^b^ ± 3951	6478 ^a^ ± 4154
Photo ^(NS)^	4119 ^c^ ± 3994	3771 ^c^ ± 4440	5209 ^b^ ± 4005	3522 ^b^ ± 3761
Type of wine ^(NS)^	7674 ^ab^ ± 3092	8447 ^ab^ ± 3056	7394 ^ab^ ± 3462	7730 ^a^ ± 3323
Volume ^(NS)^	9137 ^a^ ± 2111	9469 ^a^ ± 1689	9650 ^a^ ± 1362	8861 ^a^ ± 2249
Year ^(NS)^	9038 ^a^ ± 2309	8214 ^a^ ± 3162	7475 ^ab^ ± 3537	8597 ^a^ ± 2537

* ^A,B^ For each area of interest, means with different capital letters within a row indicate significant differences (*p* < 0.05) between treatment labels. ^NS^ For non-significant differences (*p* > 0.05) within a row. ^a–c^ For each treatment label, means with different lower case letters within a column indicate significant differences (*p* < 0.05) between areas of interest.

**Table 2:** Means and standard deviations of the first fixation duration (ms) on each AOI for four labels *.

Area of Interest	COO Information			
	Script	No Script	Logo 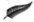	No Logo
Alcohol % ^(NS)^	20.4 ^b^ ± 40.5	31.2 ^bc^ ± 45.2	23.0 ^ab^ ± 44.4	35.1 ^b^ ± 61.8
COO ^(NS)^	18.9 ^b^ ± 45.7	1.5 ^c^ ± 13.1	30.3 ^ab^ ± 54	19.0 ^b^ ± 46.8
Brand name ^(NS)^	46.5 ^ab^ ± 53.2	42.1 ^ab^ ± 48.1	57.3 ^a^ ± 50.9	54.3 ^ab^ ± 65.7
Photo ^(NS)^	63.6 ^a^ ± 46.3	73.9 ^a^ ± 58.4	57.8 ^a^ ± 50.7	84.1 ^a^ ± 47.8
Type of wine ^(NS)^	36.4 ^ab^ ± 42.5	26.8 ^bc^ ± 50.3	43.6 ^ab^ ± 62.8	36.4 ^b^ ± 62.7
Volume ^(NS)^	18.7 ^b^ ± 41.5	17.6 ^bc^ ± 41.1	6.9 ^b^ ± 30.7	29.2 ^b^ ± 56.6
Year ^(NS)^	22.1 ^b^ ± 53.2	29.1 ^bc^ ± 56.5	43.8 ^ab^ ± 58.3	19.1 ^b^ ± 36.2

* ^NS^ For non-significant differences (*p* > 0.05) within a row between treatment labels. ^a–c^ For each treatment label; means with different lower case letters within a column indicate significant differences (*p* < 0.05) between areas of interest.

**Table 3:** Means and standard deviations of the average fixation duration (ms) on each AOI for four labels *.

Area of Interest	COO Information			
	Script	No Script	Logo 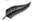	No Logo
Alcohol % ^(NS)^	21.0 ^b^ ± 41.5	31.2 ^bc^ ± 45.2	24.2 ^ab^ ± 46	35.1 ^ab^ ± 61.8
COO	19.1 ^A,b^ ± 46.1	1.5 ^B,c^ ± 13.1	30.3 ^A,ab^ ± 54	19.0 ^A,b^ ± 46.8
Brand name ^(NS)^	49.4 ^ab^ ± 54.5	42.1 ^ab^ ± 48.1	56.7 ^a^ ± 48.5	54.3 ^ab^ ± 65.7
Photo ^(NS)^	71.8 ^a^ ± 48.5	73.9 ^a^ ± 58.4	59.3 ^a^ ± 51.5	84.1 ^a^ ± 47.8
Type of wine ^(NS)^	37.7 ^ab^ ± 43.6	26.8 ^bc^ ± 50.3	41.5 ^ab^ ± 57.1	36.4 ^b^ ± 62.7
Volume	20.1 ^A,b^ ± 44.4	17.6 ^A,bc^ ± 41.1	6.9 ^B,b^ ± 30.7	29.2 ^A,b^ ± 56.6
Year ^(NS)^	20.4 ^b^ ± 48.9	29.1 ^bc^ ± 56.5	40.9 ^ab^ ± 54.5	19.1 ^b^ ± 36.2

* ^A,B^ For each area of interest, means with different capital letters within a row indicate significant differences (*p* < 0.05) between treatment labels. ^NS^ For non-significant differences (*p* > 0.05) within a row. ^a–c^ For each treatment label, means with different lower case letters within a column indicate significant differences (*p* < 0.05) between areas of interest.

**Table 4:** Means and standard deviations of the fixation count on each AOI for four labels *.

Area of Interest	COO Information			
	Script	No Script	Logo 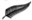	No Logo
Alcohol % ^(NS)^	0.3 ^b^ ± 0.6	0.6 ^b^ ± 1 b	0.3 ^bc^ ± 0.5	0.8 ^b^ ± 1.5
COO ^(NS)^	0.6 ^b^ ± 1.6	0.0 ^b^ ± 0.2	0.3 ^bc^ ± 0.5	0.2 ^b^ ± 0.4
Brand name ^(NS)^	1.1 ^b^ ± 1.5	1.6 ^b^ ± 2	1.6 ^b^ ± 1.6	1.2 ^ab^ ± 1.7
Photo ^(NS)^	3.9 ^a^ ± 3.7	5.1 ^a^ ± 4.9	3.4 ^a^ ± 4.2	4.4 ^a^ ± 3.3
Type of wine ^(NS)^	0.9 ^b^ ± 1.1	0.6 ^b^ ± 1.2	0.9 ^bc^ ± 1.3	1.1 ^b^ ± 1.9
Volume ^(NS)^	0.3 ^b^ ± 0.5	0.2 ^b^ ± 0.5	0.1 ^c^ ± 0.3	0.3 ^b^ ± 0.5
Year ^(NS)^	0.2 ^b^ ± 0.5	0.3 ^b^ ± 0.6	0.7 ^bc^ ± 1.0	0.8 ^b^ ± 1.7

* ^NS^ For non-significant differences (*p* > 0.05) within a row between treatment labels. ^a–c^ For each treatment label, means with different lower case letters within a column indicate significant differences (*p* < 0.05) between areas of interest.

## Data Availability

The data presented in this study are available on request from the corresponding author.

## Supplementary Materials

The following are available online at https://www.mdpi.com/article/10.3390/s22062158/s1, Table S1: Cochran’s Q test results of the CATA emotions frequencies (study 2); Table S2: Cochran’s Q test results of the CATA sensory frequencies (study 2).
