# Supplementary material for: Influence of Label Design and Country of Origin Information in Wines on Consumers’ Visual, Sensory, and Emotional Responses
Source: Sensors (Basel). 2022 Mar 10;22(6):2158. doi: 10.3390/s22062158 (PMC8949006; doi:10.3390/s22062158)
Supplement: Supplementary file 1 [file sensors-22-02158-s001.zip › sensors-1584146-supplementary.pdf]

**Table S1** Cochran's Q test results of the CATA emotions frequencies (study 2)

| Attributes | Baseline | Script | No script | Logo | No Logo |
|---|---|---|---|---|---|
| Happy | 0.23 a | 0.29 a | 0.34 a | 0.26 a | 0.27 a |
| Neutral | 0.39 b | 0.27 b | 0.45 a | 0.16 b | 0.32 b |
| Sad | 0.01 a | 0.00 a | 0.05 a | 0.05 a | 0.03 a |
| Curious | 0.36 a | 0.24 a | 0.24 a | 0.34 a | 0.27 a |
| Disgusted | 0.02 a | 0.03 a | 0.08 a | 0.08 a | 0.03 a |
| Surprised | 0.13 a | 0.12 a | 0.054 a | 0.08 a | 0.15 a |
| Excited | 0.18 a | 0.12 a | 0.24 a | 0.16 a | 0.12 a |
| Pleased | 0.28 a | 0.35 a | 0.40 a | 0.37 a | 0.32 a |
| Calmed | 0.25 a | 0.21 a | 0.32 a | 0.24 a | 0.29 a |
| Apprehensive | 0.07 a | 0.06 a | 0.05 a | 0.21 a | 0.12 a |
| Comforted | 0.27 a | 0.29 a | 0.26 a | 0.34 a | 0.27 a |
| Satisfied | 0.27 a | 0.18 a | 0.18 a | 0.34 a | 0.15 a |
| Bored | 0.04 a | 0.09 a | 0.11 a | 0.08 a | 0.09 a |
| Guilty | 0.03 a | 0.00 a | 0.03 a | 0.05 a | 0.03 a |
| Healthy | 0.18 a | 0.18 a | 0.16 a | 0.11 a | 0.06 a |
| Unhealthy | 0.07 a | 0.06 a | 0.00 a | 0.03 a | 0.09 a |

If the groups share the same lowercase letters indicate no statistically significant difference ($p \geq 0.05$) between the treatment labels (rows). Baseline = no label.

**Table S2** Cochran's Q test results of the CATA sensory frequencies (study 2)

| Attributes | Baseline | Script | No script | Logo | No Logo |
|---|---|---|---|---|---|
| Processed fruit flavour | 0.12 a | 0.09 a | 0.16 a | 0.32 a | 0.24 a |
| Green flavour | 0.11 a | 0.15 a | 0.11 a | 0.05 a | 0.12 a |
| Spicy flavour | 0.22 a | 0.06 a | 0.24 a | 0.32 a | 0.06 a |
| Earthy flavour | 0.09 a | 0.18 a | 0.13 a | 0.11 a | 0.12 a |
| Chemical flavour | 0.08 ab | 0.18 a | 0.18 a | 0.16 a | 0.00 b |
| Heat feeling | 0.36 a | 0.15 b | 0.18 b | 0.26 b | 0.12 b |
| Sweet taste | 0.15 b | 0.15 b | 0.26 b | 0.24 b | 0.38 a |
| Bitter taste | 0.36 a | 0.41 a | 0.37 a | 0.32 a | 0.29 a |
| Astringent | 0.51 a | 0.38 a | 0.58 a | 0.53 a | 0.27 a |
| Sour taste | 0.43 a | 0.47 a | 0.37 a | 0.47 a | 0.32 a |
| Floral | 0.11 a | 0.12 a | 0.26 a | 0.29 a | 0.18 a |
| Fruity flavour | 0.45 a | 0.32 a | 0.34 a | 0.42 a | 0.35 a |

If the groups share the same lowercase letters indicate no statistically significant difference ($p \geq 0.05$) between the treatment labels (rows). Baseline = no label.
